# Lack of Male-Female Differences in Disposition and Esterase Hydrolysis of Ramipril to Ramiprilat in Healthy Volunteers after a Single Oral Dose

**DOI:** 10.1100/tsw.2003.122

**Published:** 2003-12-11

**Authors:** Tom B. Vree, Erik Dammers, Ivan Ulc, Stefan Horkovics-Kovats, Miroslav Ryska, IJsbrand Merkx

**Affiliations:** ^1^ Institute for Anaesthesiology, University Medical Centre Nijmegen Sint Radboud, Nijmegen, The Netherlands; ^2^ DADA Consultancy, Nijmegen, The Netherlands; ^3^ Cepha s.r.o., Pilsen, Czech Republic; ^4^ Biochemie GmbH, Kundl, Austria; ^5^ Quinta-Analytica, Prague, Czech Republic; ^6^ Novartis, Weesp, The Netherlands

**Keywords:** ramipril, ramiprilat, pharmacokinetics, liver, gut, hydrolysis, plasma concentration, male-female, elimination half-life

## Abstract

The objective of this study was to identify differences in disposition and esterase hydrolysis of ramipril between male and female volunteers. Plasma concentration and area under the concentration-time curve until the last measured concentration (AUCt) data of ramipril and its active metabolite ramiprilat (-diacid) were obtained from a randomised, cross-over bioequivalence study in 36 subjects (18 females and 18 males). Participants received a single 5-mg oral dose of two different formulations of ramipril (Formulation I and II). Plasma ramipril and ramiprilat concentrations were determined according to validated methods involving liquid chromatography-mass spectrometry. A total number of 2 × 34 available plasma concentration-time curves of both the parent drug and the metabolite could be analysed, and variations (50—100% coefficient of variation [CV]) in plasma concentrations of both parent drug and metabolite were found. With both the formulations, the mean plasma concentrations-time curves of males and females were identical. The groups of female and male volunteers showed similar yields (AUCt = mg.h/L) of the metabolite ramiprilat (p = 0.37); however, females showed a higher AUCt/kg than males (p = 0.046). This difference was solely attributed to the difference in body weight between males and females (p = 0.00049). In both male and female groups, a subject-dependent yield of active metabolite ramiprilat was demonstrated, which was independent of the formulation.There is a large variation in the ramiprilat t_1/2β_ (50—60% CV). There is a group of subjects who showed a t_1/2β_ of approximately 80 h (15% CV), and two apparent groups with a longer t_1/2β_for each formulation (124 h, 22.5% CV; 166 h, 21.6% CV, respectively, p = 0.0013). This variation in the terminal half-life of ramiprilat is not sex related. In all three groups of half-lives, the corresponding Cmax values (mean ± SD) of ramiprilat in males and females were identical. Thus, with identical C_max_ and half-lives, the difference found in the AUC_t_ /kg of ramiprilat must be due to the difference in dose, as the consequence of the difference in body weight, following a standard dose of 5 mg in both males and females.This study showed clearly that despite subject-dependent hydrolysis of ramipril to the active metabolite ramiprilat, the variability in the rate of hydrolysis between males and females is similar. With a fixed dose (5 mg), females received a higher dose/kg than males and consequently showed a higher AUC_t_/kg of the active metabolite ramiprilat.

## INTRODUCTION

RAMIPRIL is a member of the second generation of angiotensin-converting enzyme (ACE) inhibitors[1,2,3]. It is a pro-drug that is hydrolysed *in vivo* releasing the active metabolite ramiprilat[4,5]. The metabolite has a long elimination half-life (≈100 h) permitting once-a-day administration[1,2]. Ramiprilat binds to ACE with very high affinity, and is active at concentrations similar to those of the enzyme, establishing the equilibrium slowly[6].

The clinical data confirm ramipril as a useful ACE-inhibitor alternative for the treatment of patients with mild to moderate hypertension, with an indication of the beneficial effect in patients with clinical evidence of heart failure after acute myocardial infarction. Ramipril is also supposed to be valuable in the treatment of patients with more established heart failure of asymptomatic left ventricular dysfunction. When administered to patients with essential hypertension, a single oral dose of 2.5–10 mg decreased systolic and diastolic blood pressure in a dose-dependent manner without affecting the normal circadian variation in blood pressure or heart rate[2].

Following administration of 5 mg of ramipril, the maximum inhibition of ACE activity in plasma of 98.2 ± 2.18% was observed after 2.74 ± 1.42 h (mean ± SD). The relationship between the concentrations of ramiprilat and the inhibition of ACE activity subsequently declined in an exponential manner. The duration of inhibition >80% of plasma ACE activity was 16.8 ± 4.47 h. The half-maximum effect occurred at a plasma concentration of about 1 ng/mL of ramiprilat; at a concentration of 2 ng/mL, ACE activity was inhibited by more than 80%[7,8]. Enzyme activity was still inhibited by 40–50% at 72 h after the dose of ramipril[8,9]. In the treatment of mild to moderate essential hypertension, the initial recommended oral dose of ramipril is normally 2.5 mg once daily. The dosage should be titrated according to the patient’s blood pressure response, and the usual maintenance dosage is 2.5 or 5 mg/d, with a maximum daily dose of 10 mg[3,10].

A bioequivalence study with 36 subjects (males/females) and a dose of 5 mg ramipril revealed subject-related differences in the absorption, metabolism, and elimination of ramipril and its primary active metabolite, ramiprilat. The fixed dose for each subject resulted in different doses when expressed as dosage/body weight.

The aim of this study was to investigate whether the parameters of sex and body weight cause great variation in the part of the ramipril dose that is hydrolysed by esterase activity, using the plasma concentration-time curves of ramipril and its primary active metabolite.

## METHODS

### Experimental Design

The evaluation was based on the data of plasma concentration-time curves obtained from a single centre, open, randomised, two-way, crossover bioequivalence study on ramipril 5-mg tablet (Biochemie GmbH,

Kundl, Austria) vs. Delix® 5 mg (Hoechst AG, Haar, Germany) involving 36 healthy Caucasian volunteers without any comedication.

The clinical trial was conducted by Cepha s.r.o. (Pilsen, Czech Republic) after being granted approval by the Institutional Ethics Board and after informed consent was obtained from the subjects.

## Subjects

Thirty-six healthy Caucasian subjects (from the Charles University, Medical School, Pilsen), 18 males and 18 females (nonpregnant, nonbreastfeeding) participated in the study. The demographic data are summarised in Table 1.

### *Inclusion Criteria*

Age: 18–45 years, body-mass index 19–28 kg/$m^2$, physical examination without significant deviations, vital signs and ECG without significant deviations, all laboratory screening results within the normal range or being assessed as nonsignificant by the attending physician.

### *Exclusion Criteria*

- History of severe allergy or allergic reactions to study or related drug
- History of serious clinical illness that can impact fate of drugs
- Clinically significant illness within 4 weeks before study period 1
- Any significant clinical abnormality including HBsAg, HIV positivity
- Sitting blood pressure after 5 min rest lower than 100/50 mmHg
- Pregnancy, breastfeeding
- Serious mental disease, drug, alcohol, solvents or caffeine abuse, smoking more than ten cigarettes per day
- Regular use of medication except oral contraceptive steroids (OCS) taken without changes for 3 months at least
- Use of organ toxic drugs within 3 months before study period 1
- Systemic treatment by drugs (1) inducing hepatic metabolism, (2) similar to the investigational drug within 30 d before study period 1
- Any systemic prescription drug treatment within 14 d before study period 1
- Any systemic over-the-counter (OTC) drug treatment within 7 d before study period 1
- Donation of (1) at least 400 mL of blood within 60 d, (2) more than 150 mL of blood within 30 d, or (3) blood plasma or platelets within 14 d before study period 1
- Participation in another clinical trial within 60 d prior to the start of study period 1

**TABLE 1**
**Comparison Between the Pharmacokinetic Parameters of Ramipril and Ramiprilat (Formulation I + II) in Males and Females**

| | **Males** | **Females** | ***p*** | **All** |
|---|---|---|---|---|
| Dose, 5 mg | | | | |
| N | 2 × 16 | 2 × 18 | | |
| Age, year | 26.8 ± 5.9 | 26.9 ± 8.3 | 0.99 | |
| Height, cm | 180 ± 6.9 | 169 ± 4.4 | **<0.0001** | |
| Body weight, kg | 79.5 ± 11.8 | 65.7 ± 7.7 | **0.00049** | |
| Dose, μg/kg | 64.4 ± 10.6 | 77.1 ± 8.7 | **0.00047** | |
| **Ramipril** | | | | |
| $AUC_t$, μg.h/L | 10.3 ± 7.76 | 10.5 ± 6.37 | 0.91 | 10.4 ± 7.09 |
| $AUC_t$/kg, μg.h/L/kg | 0.131 ± 0.093 | 0.157 ± 0.009 | 0.10 | 0.143 ± 0.009 |
| $t_{1/2\ abs}$, h | 0.08 ± 0.12 | 0.07 ± 0.07 | 0.67 | 0.08 ± 0.09 |
| $t_{1/2\alpha}$, h | 0.26 ± 0.17 | 0.20 ± 0.08 | 0.06 | 0.23 ± 0.14 |
| $t_{1/2\beta}$, h | 1.27 ± 0.75 | 1.32 ± 1.13 | 0.83 | 1.30 ± 1.94 |
| MRT, h | 1.24 ± 0.49 | 1.11 ± 0.55 | 0.31 | 1.18 ± 0.52 |
| $t_{lag}$, h | 0.16 ± 0.02 | 0.16 ± 0.02 | 1.00 | 0.16 ± 0.02 |
| $t_{max,}$, h | 0.34 ± 0.17 | 0.30 ± 0.06 | 0.19 | 0.32 ± 0.13 |
| $C_{max}$, μg/L | 12.7 ± 5.90 | 16.0 ± 6.19 | **0.028** | 14.3 ± 6.20 |
| **Ramiprilat** | | | | |
| $AUC_t$, μg.h/L | 137 ± 33.8 | 130 ± 28.3 | 0.37 | 134 ± 31.0 |
| $AUC_t$/kg, μg.h/L/kg | 1.75 ± 0.44 | 1.97 ± 0.45 | **0.046** | 1.85 ± 0.45 |
| $t_{1/2\ abs}$, h | 2.06 ± 0.76 | 2.42 ± 0.96 | 0.09 | 2.24 ± 0.86 |
| $t_{1/2\alpha}$, h | 2.69 ± 2.18 | 2.93 ± 1.26 | 0.58 | 2.80 ± 1.78 |
| $t_{1/2\beta}$, h | 118 ± 66.1 | 104 ± 33.5 | 0.97 | 111 ± 53.2 |
| MRT, h | 158 ± 94.0 | 133 ± 42.4 | 0.16 | 145 ± 74.5 |
| $t_{lag}$ h | 0.13 ± 0.16 | 0.10 ± 0.03 | 0.27 | 0.11 ± 0.11 |
| $t_{max}$, h | 4.25 ± 1.45 | 4.61 ± 1.69 | 0.35 | 4.33 ± 1.54 |
| $C_{max}$, μg/L | 4.14 ± 1.67 | 4.29 ± 1.50 | 0.70 | 4.18 ± 1.57 |

## *Trial Course*

Participants were divided randomly into two groups with the aid of a computer-generated randomisation list. Group 1 was assigned to treatment sequence I-II (Formulation I and II). Group 2 was assigned to sequence II-I. During the cross-over sessions, volunteers received each of the following treatments after an overnight fast, administered with 240 mL of water:

- Formulation I: single oral dose of one ramipril 5-mg tablet, batch number 276 (Ramipril, Biochemie GmbH, Kundl, Austria)
- Formulation II: single oral dose of one ramipril 5-mg tablet, batch number 40C503 (Delix®, Hoechst, Germany)

## Drugs

- Ramipril (C23H32N2O5, MW 416.22, [2S-[1[R*(R*)], 2α, 3aβ, 6aβ]]-1-[2 [[1-(**ethoxycarbonyl**)-3-phenylpropyl]amino]-1-oxopropyl]octahydrocyclopenta [b]pyrrole–2-carboxylic acid, CAS - 87333-19-5 and
- Ramiprilat C21H28N2O5, MW 388.52, [2S-[1[R*(R*)], 2α, 3aβ, 6aβ]]-1-[2 [[1-(**carbonyl**)-3-phenylpropyl]amino]-1-oxopropyl]octahydrocyclopenta[b]pyrrole-2-carboxylic acid, were obtained from Biochemie.

### *Drug Administration*

Before drug administration, participants fasted for at least 10 h, and this was continued until 4 h after drug administration. The subjects were not allowed to lie down or sleep for the first 3 h after dosing, to ensure normal absorption. If dizziness occurred, the subjects were permitted to lie down on their right side.

No alcohol-, caffeine-, xanthine- or grapefruit-containing food or drink was allowed within 72 h before each drug administration and during the confinement postdose periods.

From 48 h prior to each study period until 32 h after each study period, the intake of $CO_2$-containing beverages and smoking were prohibited. No strenuous activities were allowed 24 h before screening and follow-up examinations.

## Food and Fluid Intake

The subjects received 240 mL of water upon drug administration, and at 2 and 5 h after drug administration. Standardised meals with additional fluid (240 mL) were provided 10.5 h before drug administration and at 5 h (with 490 mL fluid), 9 h (with 240 mL), 12 h (with 240 mL), 15 h (with 240 mL), 25 h (with 240 mL), and 29 h (with 490 mL) after drug administration. The subjects were free to drink additional supplied beverages free of alcohol, $CO_2$, caffeine, and grapefruit from 6 h after drug administration.

## Blood Sampling

On the day of drug administration, an indwelling intravenous cannula (Vasocan Braunüle 20 G 11/4") was inserted in a forearm vein of each volunteer. The cannula was removed after withdrawal of the 16-h postadministration sample. The remaining blood samples (24–96 h) were drawn by venepuncture.

Blood samples (8 mL) were collected in polypropylene test tubes, containing 0.20 mL of 5% $Na_2EDTA$ as anticoagulant, just before administration and at 0.17, 0.50, 0.75, 1, 1.33, 1.67, 2, 3, 4, 5, 6, 8, 10, 12, 16, 24, 32, 48, 72, and 96 h after administration.

The blood samples were shaken gently, and centrifuged within 15 min of collection at 4000 rpm (2000 *g*) for 10 min at 4$^o$C. The plasma samples were collected in three splits, capped, flash frozen and kept on dry ice until their storage in the freezer at –25 ± 5$^o$C. The frozen plasma samples were stored in a freezer with $CO_2$ back-up at –25 ± 5$^o$C until their transportation on dry ice to the bioanalytical facility of Quinta-analytica and stored thereafter at –25 ± 5$^o$C until analysis.

## Bioanalysis

Ramipril and its metabolite ramiprilat were analysed by means of high performance liquid chromatography-mass spectrometry (LC-MS, Quinta-analytica s.r.o., Prague, Czech Republic).

***In brief:***

*Chemicals*

- Ramipril $C_{23}H_{32}N_2O_5$, MW 416.52; Ramiprilat $C_{21}H_{28}N_2O_5$, MW 388.47

*Internal standards*

- Enalapril $C_{20}H_{28}N_2O_5$, MW 376.45 (Maleate salt, batch no 038H0500, Sigma)
- Enalaprilat $C_{18}H_{24}N_2O_5$, MW 348.40 (Quinta-Analytica)
- Chemicals (pro-analysis) were obtained from Baker, Merck, Fluka, Sigma, and Novartis.

## Instrumentation

RHEOS 2000 (Flux Instrument) HPLC, with a MS detector (TSQ, ThermoQuest/Finnigan), equipped with a column: TPR-100, 5 μm (3.0 × 20 mm, Supelco). Injection: 15 μL. Acquisition; ESI mode, positive polarity, scan MS/MS.

Mobile phase: acetonitrile, methanol, 2% formic acid, and water, in a program mode during 4.5 min, and a flow rate varying between 350 and 1200 μL/min.

The mass-chromatograms reconstructed from the ion profiles corresponding to ramipril (*m/z* 233.5), ramiprilat (*m/z* 205.5), enalapril (*m/z* 233.5) and enalaprilat (*m/z* 205.5) have been integrated and the ratios ramipril/enalapril and ramiprilat/enalaprilat were used for the calculation of the concentrations of ramipril and ramiprilat. Retention time of the analytes was 1.1 min.

## Sample Treatment

0.05 mL of internal standard solutions (10 μg/mL enalapril and enalaprilat), 0.05 mL of water, or 0.05 mL of the appropriate working solutions of ramipril and ramiprilat, 1.0 mL of drug free plasma and 0.15 mL of 10% formic acid in water were pipetted into test tubes. Blank samples were without working and internal standard solutions. All samples were homogenised by vortexing for 5 s, and put into a freezer (–25°C) for further processing.

Before processing, the samples were thawed. After centrifugation, the samples were transferred onto extraction columns and the analytes were isolated from plasma by a solid-phase extraction procedure. After extraction the samples were evaporated to dryness, the residues were dissolved in 0.2 mL of 25% acetonitrile with 5% formic acid and then analysed using a HPLC/MS system.

## Performance

### Ramipril

The linear response was observed in the ramipril/enalapril peak area ratio over the concentration range 0.2–40 ng/mL (r = 0.999). The Lower Limit of Quantification (LOQ) was 0.2 ng/mL (3.1% CV) at a signal to noise ratio of 5.

The *intra-assay* precision was 3.4% with the accuracy –0.7%, at a concentration of 0.20 ± 0.007 ng/mL. These data were 3.9% precision and –1.8% accuracy at 39.3 ± 1.54 ng/mL.

The *inter-assay* precision was 3.4% with the accuracy –0.7%, at a concentration of 0.20 ± 0.007 ng/mL. These data were 1.1% precision and –1.8% accuracy at 40.2 ± 0.43 ng/mL.

### *Ramiprilat*

The linear response was observed in the ramiprilat/enalaprilat peak area ratio over the concentration range 0.2–20 ng/mL (r = 0.999). The Lower Limit of Quantification (LOQ) was 0.2 ng/mL (3.1% CV) at a signal to noise ratio of 5.

The *intra-assay* precision was 7.4% with the accuracy –4.4%, at a concentration of 0.19 ± 0.014 ng/mL. These data were 2.7% precision and –1.6% accuracy at 19.7 ± 0.54 ng/mL.

The *inter-assay* precision was 7.4% with the accuracy –4.4%, at a concentration of 0.20 ± 0.007 ng/mL. These data were 1.8% precision and –0.5% accuracy at 19.9 ± 0.35 ng/mL.

## Pharmacokinetic Analysis

Based on plasma ramipril and ramiprilat concentrations of individual volunteers, the following pharmacokinetic parameters were determined by noncompartmental and two-compartmental analysis using the program MW/Pharm[11].

- Maximum plasma concentration ($C_{max}$, μg/L); time to reach peak concentration ($t_{max}$, h)
- Area under the plasma concentration-time curve until the time of the last measured concentration ($C_t$), calculated by the linear trapezoidal rule ($AUC_t$, μg.h/L)
- $AUC_t$/kg is the $AUC_t$ corrected for the body weight
- Elimination half-life ($t_{1/2\beta}$) associated with the terminal slope of a semilogarithmic concentration-time curve ($t_{1/2z}$, h) calculated as $\ln 2/\lambda_z$, where $\lambda_z$ is the elimination rate constant
- Lag time ($t_{lag}$) was defined as the time between administration and first measurable plasma concentration
- $t_{1/2absorption}$ was the absorption half-life
- $t_{1/2\alpha}$ was the half-life of the rapid elimination phase
- Mean residence time (MRT) = $AUCM_{0-\infty}/AUC_{0-\infty}$, where $AUC_{0-\infty}$ was the area under the moment curve from zero to infinity

## Statistical Analysis

Analysis of variance (ANOVA, two-tailed, Gaussian distribution) of the pharmacokinetic parameters was carried out and significance was defined at $p < 0.05$.

## RESULTS

A total number of 2 × 34 available plasma concentration-time curves of both the parent drug and the metabolite were analysed, and variations (50–100% CV) in plasma concentrations of both parent drug and metabolite were found. With both the formulations, the mean plasma concentrations-time curves of males and females were identical.

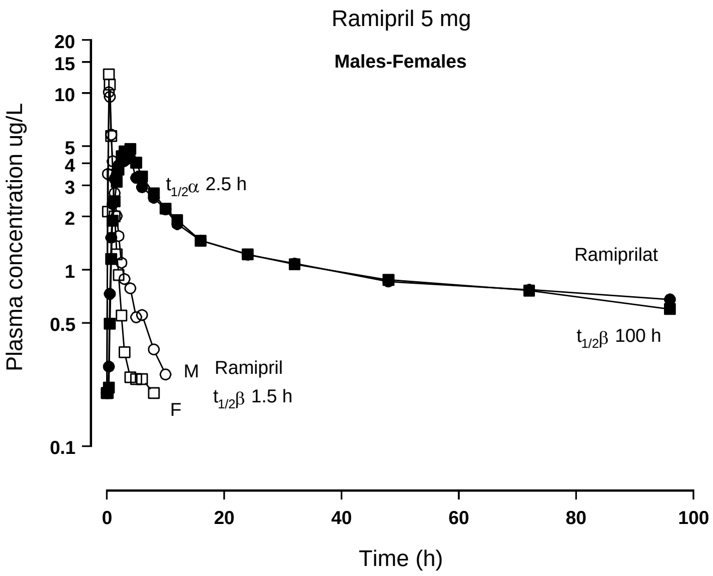

**FIGURE 1.** Mean plasma concentration-time curves of ramipril (open markers) and its metabolite, ramiprilat (solid markers), in 16 males (M, dots) and 18 females (F, squares) after the oral administration of 5 mg ramipril (Formulation I). Males and females show identical mean plasma concentration curves. Similar results were obtained when after administration of Formulation II. SD bars are omitted for reasons of clarity. The 50% ACE activity is present at 1 ng/mL (1 μg/L).

The exemplary mean plasma concentration-time curves of males and females with Formulation I are given in Fig. 1.

The pharmacokinetic parameters of ramipril and ramiprilat in males and females are shown in Table 1 after administration of Formulation I + II. The demographic parameters body weight, and body length show statistical significant differences between males and females. The pharmacokinetic parameters of ramipril and ramiprilat in males and females were identical.

**Ramipril pharmacokinetics** — ramipril is rapidly absorbed from the gastrointestinal tract, as shown by the small lag-time of 0.16 ± 0.02 h, a small $t_{1/2absorption}$ of 0.08 ± 0.09 h, the small $t_{max}$ of 0.32 ± 0.13 h, and the $C_{max}$ of 14.3 ± 6.2 mg/L (all subjects). After absorption. ramipril was eliminated following a two compartment-model (r = 0.999) with a $t_{1/2\alpha}$ of 0.23 ± 0.14 h and a $t_{1/2\beta}$ of 1.30 ± 1.94 h. A perfect two-compartment elimination kinetics after oral administration was obtained in the individual curves when the ratio $t_{1/2absorption}$:$t_{1/2\alpha}$:$t_{1/2\beta}$ is 1:5:25. This ratio was present in 53 administrations, but was 1:1:5 in 15 administrations (7M, 8F).

**Ramiprilat pharmacokinetics** — ramiprilat is rapidly formed from the gastrointestinal tract, as shown by the small lag-time of 0.11 ± 0.11 h, a small $t_{1/2absorption}$ of 2.24 ± 0.81 h, the $t_{max}$ of 4.33 ± 1.54 h, and the $C_{max}$ of 4.18 ± 1.57 mg/L (all subjects). After formation, ramiprilat was eliminated following a two compartment-model (r = 0.999) with a $t_{1/2\alpha}$ of 2.80 ± 1.78 h and a $t_{1/2\beta}$ of 111 ± 53.2 h. A perfect two-compartment elimination kinetics after oral administration was obtained in the individual curves when the ratio $t_{1/2absorption}$:$t_{1/2\alpha}$:$t_{1/2\beta}$ is 1:1:50. This ratio was present in 54 administrations (26F, 28M), but was 0.1:5:50 in 14 administrations (6M, 8F).

The deviation from the ideal ratio of the rate constants in the majority of the subjects in ramipril and ramiprilat was not related to the subjects, only 4 subjects (3M, 1F) (13.8%, out of 15 ramipril and 14 ramiprilat ratio deviations) showed deviations in both compounds.

It seems that $C_{max}$ and $t_{1/2}$ values are independent parameters, the sex ratio is evenly spread over the groups based on these two parameters.

There is a large variation in the $t_{1/2\beta}$ (50–60% CV). There is a group of subjects who showed a $t_{1/2\beta}$ of approximately 80 h (15% CV), and an apparent second group with a longer $t_{1/2\beta}$ for each formulation (124 h, 22.5% CV; 166 h, 21.6% CV) as shown in Table 2.

**TABLE 2**
**Variation in the Terminal Elimination Half-Life (h) of Ramiprilat in Apparent Groups of Subjects (34 Subjects)**

| Formulation | I | *n* | II | *n* | *p* |
|---|---|---|---|---|---|
| *All* | 103 ± 67 h | (*34)* | 120 ± 68 h | *(34)* | 0.29 |
| *Subgroup a* | 78 ± 12 h | *(18)* | 81 ± 11 h | *(18)* | 0.42 |
| *p* | ***<0.0001*** | | ***<0.0001*** | | |
| *Subgroup b* | 124 ± 28 h | *(16)* | 166 ± 36 h | *(16)* | **0.0013** |
| The terminal half-lives (h) do not depend on the male/female sex difference. | | | | | |
| *Subgroup a* | | | | | |
| Males | 78.4 ± 11.9 | *(9)* | 77.8 ± 21.0 | *(8)* | |
| *p* | *0.63* | | *0.71* | | |
| Females | 81.4 ± 13.2 | *(9)* | 80.7 ± 11.1 | *(10)* | |
| *Subgroup b* | | | | | |
| Males | 120 ± 23.0 | *(8)* | 142 ± 21.4 | *(7)* | |
| *p* | *0.60* | | *0.28* | | |
| Females | 128 ± 32.5 | *(8)* | 188 ± 37.3 | *(9)* | |

Table 2 shows that the variation in the terminal half-life of ramiprilat is not sex related. Also, in all groups of half-lives, the corresponding $C_{max}$ values (mean ± SD) of ramiprilat in males and females were identical.

Thus, with identical $C_{max}$ and half-lives, the difference found in the $AUC_t$/kg of ramiprilat (Fig. 3) must be due to the difference in dose, as the consequence of the difference in body weight, following a standard dose of 5 mg in both males and females.

## Male-Female Differences in Ramipril Hydrolysis

Exemplary differences in $AUC_t$ of both ramipril and the active metabolite ramiprilat for males and females of Formulation II are shown in Fig. 2. In this small group of subjects no difference was found between males and females ($p = 0.93$).

When the $AUC_t$/kg of both ramipril and the active metabolite ramiprilat for males and females of all data (Formulation I + II) were plotted, as shown in Fig. 3, females showed a significant higher $AUC_t$/kg for ramiprilat than males ($p = 0.046$), while the corresponding data for ramipril were similar ($p = 0.26$).

## Intersubject Variation of Ramipril Hydrolysis

A scatter plot of datapoints of Formulation I + II is obtained when the $AUC_t$/body weight of ramipril is plotted vs. that of the metabolite in females shown in Fig. 4 and in males shown in Fig. 5. There is a tenfold variation in the ramipril data, and a threefold variation in the ramiprilat data, indicating a more or less constant variation in hydrolysis.

The $AUC_t$/kg data of ramiprilat are a factor 10 higher than those of the parent drug ramipril.

In Fig. 4, female subject 1 appeared to be a slow hydrolyser, all other subjects hydrolyse ramipril in a comparable manner. The whole group can be split into two apparent subgroups, with similar slopes of the regression lines ($p = 0.51$), but with different Y-intercepts ($p < 0.0001$).

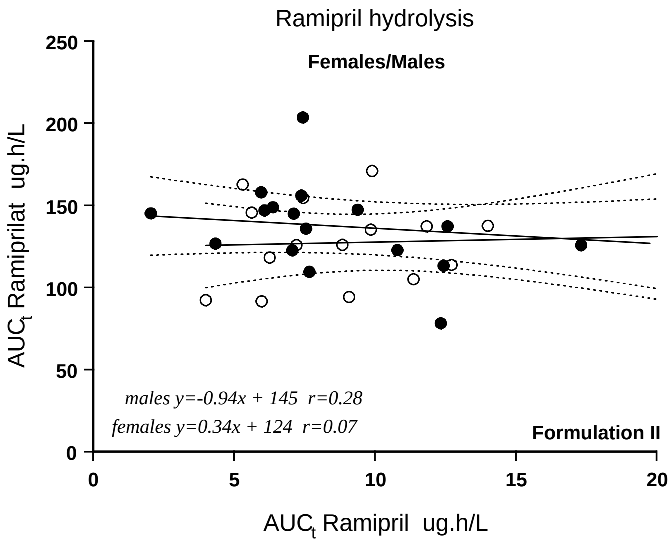

**FIGURE 2.** $AUC_t$ ramipril plotted vs. the $AUC_t$ of the metabolite ramiprilat after the oral administration of 5 mg ramipril (Formulation II) to male and female subjects. There is a 7-fold variation in the $AUC_t$ ramipril, with a 1.5-fold variation in the $AUC_t$ of the metabolite. No difference between males (solid dots) and females (open dots) in the hydrolysis of ramipril Formulation II ($p$ = 0.93). Similar results were obtained for Formulation I ($p$ = 0.93). The hydrolysis of ramipril in ramiprilat must be instantaneous, or by the gastrointestinal tract, because when extrapolating to t = 0 ($\lim X \to 0$), there is a substantial amount of ramiprilat present with no measurable $AUC_t$ of the parent drug.

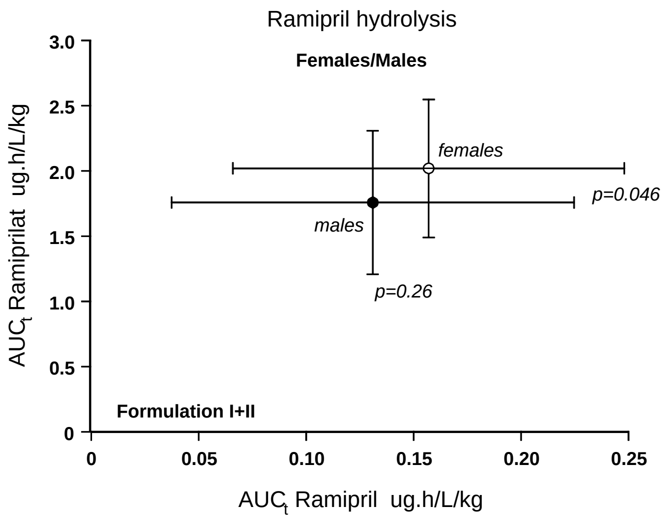

**FIGURE 3**. $AUC_t$/kg ramipril plotted vs. the $AUC_t$ /kg of the metabolite ramiprilat after the oral administration of 5 mg ramipril (Formulation I + II). There is a difference between males (solid dot, n = 32) and females (open dot, n = 36) in the hydrolysis of ramipril ($p$ = 0.046).

In Fig. 5, two male subjects (#2 and #27), appeared to be slow hydrolysers, all other subjects hydrolyse ramipril in a comparable manner. The whole group can be split into two apparent subgroups, with different slopes of the regression lines ($p$ < 0.0001), and with different Y-intercepts ($p$ = 0.0002).

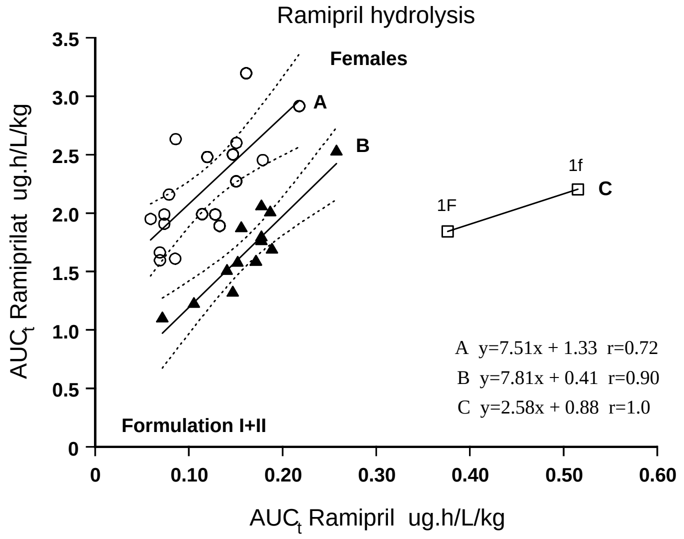

**FIGURE 4.** AUC$_t$/kg ramipril plotted vs. the AUC$_t$ /kg of the metabolite ramiprilat after the oral administration of 5 mg ramipril (Formulation I + II in females). Two apparent subgroups can be distinguished, each with a similar slope of the regression line ($p$ = 0.51) but with different Y-intercepts ($p$ < 0.0001). Subject 1 hydrolyses ramipril very slowly (F = first administration and f = second administration).

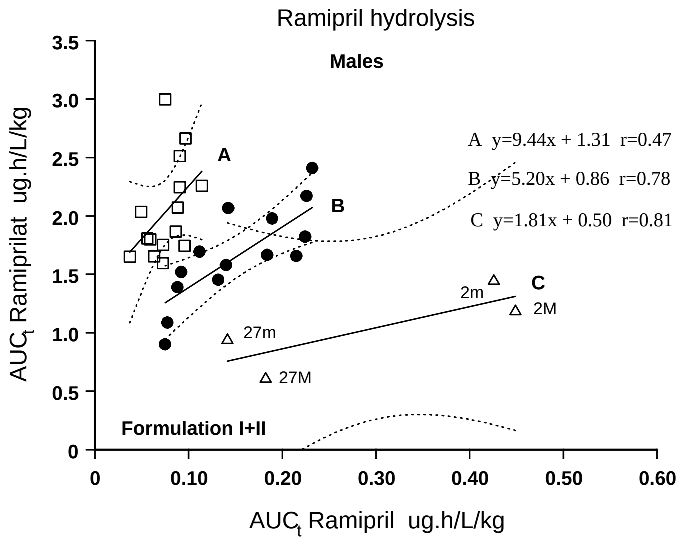

**FIGURE 5.** AUC$_t$/kg ramipril plotted vs. the AUC$_t$ /kg of the metabolite ramiprilat after the oral administration of 5 mg ramipril (Formulation I + II in males). Two apparent subgroups can be distinguished, each with a different slope of the regression line ($p$ < 0.0001) and with different Y-intercepts ($p$ = 0.0002). Subjects 2 and 27 hydrolyse ramipril very slowly (M first administration, m second administration).

There is no male-female difference between the two highest groups of Figs. 4 and 5, concerning the slopes ($p$ = 0.061) and the Y-intercepts ($p$ = 0.85). The difference is present in the lowest group, slope (F > M, $p$ < 0.0001) and Y-intercept (M > F, $p$ < 0.0001).

### Discrimination between Hydrolysis of Ramipril by the Gastrointestinal Tract and by the Plasma/Liver

When plasma and hepatic esterase hydrolysis of ramipril takes place, then the parent drug first must be absorbed and then hydrolysed. This means that with $AUC_{parent}$ approaching zero ($lim\ X \rightarrow 0$), the resulting corresponding $AUC_{metabolite}$ also must approach zero ($lim\ Y \rightarrow 0$).

In the $AUC_t$ ramipril vs. $AUC_t$ ramiprilat plot of Formulation I + II in Figs. 4 and 5, two apparent subgroups of subjects could be distinguished in the groups of males and females, each of which has a specific yield ($AUC_t$) of the metabolite ramiprilat.

There is a group of 33 administrations (15M, 18F) that renders the highest $AUC_t$ of metabolite, a group of 29 administrations (13M, 16F) that renders a low $AUC_t$, and a group of 6 administrations (4M, 2F) with the lowest $AUC_t$ (n = 68). The 95% confidence interval (CI) of the Y-intercept of the subjects who have the high and low of hydrolysis both does not contain the origin of the axis, indicating tissue hydrolysis with additional hydrolysis by liver and plasma. The 95% CI interval of the lowest hydrolysers contains the origin, indicating mainly tissue hydrolysis (low slope, low Y-intercept).

## DISCUSSION

In this study we investigated the plasma concentration-time curves and the pharmacokinetics of ramipril and its active metabolite, ramiprilat. The pharmacokinetic parameters found correspond well with those reported earlier[12,13,14].

Ramipril undergoes extensive first-pass metabolism and therefore only small amounts of unchanged ramipril can be found in urine and bile[8,15]. Both ramipril and ramiprilat are conjugated to the respective glucuronides, and the corresponding diketopiperazine derivatives are also formed[13,14,15,16]. Renal excretion is the main route of elimination of the parent drug and ramiprilat with their respective metabolites. Approximately 60% of a single oral dose of ramipril is recovered in the urine, while the remaining 40% is recovered in the faeces and includes biliary excretion, with significant enterohepatic circulation occurring in the dog, and presumably also in humans[2,7,13,17,18,19,20].

The remarkable difference between the terminal half-lives of ramiprilat in subgroups of the volunteers (80 h vs. 124–166 h) may raise the question why 50% of the subjects show this relatively "fast" elimination of 80 h and the other 50% a wide variety in extreme long half-lives. If there is no enterohepatic recirculation of ramiprilat glucuronide, the half-life of 80 h may represent the dissociation kinetics of the ACE-ramiprilat complex[21]. Interindividual variation in the enterohepatic recirculation of ramiprilat glucuronide may add to this interindividual (independent of sex) variation in the terminal half-life[22].

Another possibility may be that the variation in polymorph ACE-genes and variation in subsequent binding constants may play a role in the terminal half-life variation[23].

This study revealed a subject-dependent rate of formation of the active metabolite ramiprilat, in both females and males, which was independent of the formulation. Females showed a higher $AUC_t$/kg of ramiprilat than males, due to the higher dose/kg. The $AUC_t$ values of ramiprilat of both females and males were similar, due to the big variation in the long terminal half-life. As shown earlier for the acetylation of mesalazine and the hydrolysis of simvastatin and lovastatin by the liver and by the gastrointestinal tract, it was possible to discriminate between hydrolysis of ramipril by plasma/hepatic or tissue esterase activity[24,25,26]. When only plasma/liver esterase activity is responsible for the hydrolysis of ramipril, with the $AUC_t$/kg for ramipril approaching zero ($lim\ X \rightarrow 0$), the $AUC_t$/kg for the metabolite must also approach zero ($lim\ Y \rightarrow 0$), and the 95% confidence interval of the Y-intercept must contain the zero.

With the combined data of Formulation I and II in both females and males, three subgroups could be distinguished which showed the gradual importance of plasma/liver hydrolysis in addition to tissue hydrolysis. When this is the case, both parent drug and intestinally formed metabolite are absorbed, resulting in a measurable $AUC_t$/kg of the metabolite when the $AUC_t$/kg of ramipril is zero. Tang and Kalow first investigated the nature of carboxyesterases and reported the existence of three esterases in man, one in plasma and two different in the liver[27].

## Clinical Implications

This study showed clearly that females showed a higher yield of active metabolite ($AUC_t$/kg) than males, independent of the formulation. This difference after a fixed oral dose of 5 mg ramipril was caused by the lower body weight of the females. Fixed dose administration is common practice due to the available pharmaceutical formulations, while in the ideal situation the dose must be based on dose/kg or titrated to the required clinical effect. Both males and females showed three subgroups of subjects with high, medium, and low hydrolysis. The high metabolite yield can be attributed to plasma esterase plus hepatic microsomes and cytosol carboxyesterase activity. As a correlation between plasma concentration and enzyme activity of ACE inhibitory activity was demonstrated, the variation in plasma concentration indicates a probable variation in inhibitory activity and thus in effect[8,9]. The higher dose/kg in females may induce earlier the incidence of coughing than in males[28,29,30,31]. The effect may also vary due ACE gene polymorphism[24]. Ideally, the plasma concentration of ramipril and ramiprilat must be monitored and correlated with dose and clinical effect. When this is not feasible, the dose-clinical effect relationship should be titrated and monitored frequently when starting the therapy.

## BIOSKETCHES

**Tom B. Vree, Ph.D.,** Chemist and Pharmacologist at the University Medical Center Nijmegen, Nijmegen, Netherlands. His fields of interest include: drug analysis, drug metabolism, and pharmacokinetics. Dr. Vree is the author and/or coauthor of 600 scientific articles and 2 books.

**Erik Dammers**, Information Pharmacist, founded DADA Consultancy in 1984. DADA Consultancy has established a solid reputation for itself as a reliable and independent partner of international pharmaceutical companies. It offers a wide range of services for information scientists in the biomedical and regulatory disciplines. It has produced more than 1500 expert reports on a wide range of medical products and has an excellent track record of more than 90% successful submissions.

**Ivan Ulc, M.D., Ph.D.,** Director, Clinical Research, at CEPHA — Center for Pharmacology and Analysis, Pilsen, Czech Republic. Dr. Ulc has 84 publications, original scientific reports on clinical drug trials submitted to the Czech Regulatory Affairs Department, Ministry of Health, and other scientific contributions mostly on pharmacokinetic studies in man.
